# Integrated metagenomic and culture-dependent profiling reveals electric shavers as selective reservoirs for multidrug-resistant opportunistic pathogens

**DOI:** 10.3389/fmicb.2026.1839764

**Published:** 2026-05-07

**Authors:** Shisheng Chen, Xing Hu, Wenjing Pan, Tongyao Chen, Xinxin Xie, Yi Zhang

**Affiliations:** 1 Department of Dermatology, The Second Affiliated Hospital and Yuying Children’s Hospital of Wenzhou Medical University, Wenzhou, China; 2 School of Laboratory Medicine and Life Science, Wenzhou Medical University, Wenzhou, China; 3 Department of Dermatology, The First Affiliated Hospital of Wenzhou Medical University, Wenzhou, China

**Keywords:** convergent evolution, electric shaver microbiome, environmental filtering, mobile genetic elements, multidrug resistance, virulence–resistance coupling

## Abstract

**Introduction:**

Personal care items are commonly viewed as passive vehicles for microbial transfer; however, the physicochemical stresses they impose may actively shape microbial persistence, community composition, and the distribution of resistance-associated determinants. Electric shavers may therefore constitute an underrecognized anthropogenic niche for the enrichment of clinically relevant antimicrobial resistance traits.

**Methods:**

We sampled electric shavers from 10 individuals at early (day 2) and mature (day 21) usage stages, generating 8 high-quality metagenomes and recovering 97 viable isolates spanning 16 bacterial species. Deep metagenomic sequencing, combined with whole-genome sequencing of 45 representative isolates, was used to resolve the ecological, functional, and evolutionary features of shaver-associated microbiomes.

**Results:**

Shaver-associated community assembly was dominated by stringent environmental filtering, which promoted the repeated enrichment of stress-adapted lineages across hosts, notably *Acinetobacter ursingii* MLST3244 and *Klebsiella pneumoniae* MLST995 and MLST23. We further identified recurrent mobile genetic element-associated resistance islands and plasmid backbones in different host cohorts, suggesting repeated selection under shared anthropogenic pressures rather than direct evidence of *de novo* convergent evolution. Importantly, viable *Klebsiella pneumoniae* isolates co-carried extended-spectrum β-lactamase genes such as *bla*_SHV_ and major virulence determinants, while metagenomic profiling detected reads assigned to *mcr-* and *tet*(X)-like gene variants at the community level, targeted PCR further confirmed the presence of these resistance determinants.

**Discussion:**

Because routine shaving can generate barrier-disrupting micro-abrasions, electric shavers may function as selective reservoirs for multidrug-resistant bacteria. Our findings reveal a previously overlooked exposure interface through which everyday personal care practices may promote the enrichment and persistence of clinically important resistance and virulence determinants.

## 1 Introduction

Human skin microbiota continuously exchanges microorganisms with the surrounding environment (Byrd et al., 2018; Chen et al., 2018), particularly on the face, which is persistently exposed to air and contact surfaces. Personal care items, such as towels and electric shavers, are frequent interfaces for microbial transfer but have traditionally been considered passive carriers rather than active microenvironments driving microbial adaptation (Yong and Calautit, 2023).

Electric shavers represent a distinctive anthropogenic niche: enclosed structures, intermittent moisture, and metallic components together create stable yet extreme ecological conditions. Even without antibiotics, these pressures may favor the persistence and enrichment of pre-adapted microbial lineages—metal ions can co-select for antibiotic resistance genes, while triclosan and quaternary ammonium compounds may promote multidrug tolerance (Paul et al., 2019; Wickham, 2022; Murray et al., 2024). Micro-abrasions caused by shaving provide entry points for opportunistic pathogens naturally present on the skin. More importantly, shaving-associated infections are clinically documented (Rawson, 2007; Shaffer et al., 2022; Thapa et al., 2024). For example, a neurosurgical site infection outbreak in a tertiary hospital involved five patients with early-onset *Serratia marcescens* infections after craniotomy (Kim et al., 2020). The same bacterium was recovered from shaving razors and brushes, and whole-genome sequencing confirmed close genetic relatedness. No further infections occurred after ceasing razor shaving, highlighting razors as overlooked sources of healthcare-associated infection.

Methodologically, shotgun metagenomics enabled high-resolution profiling of community composition, functional pathways, and resistance gene repertoires within shaver micro-niches. However, metagenomic signals may derive from non-viable cells or extracellular DNA, limiting their ability to reflect the living microbiota and active resistome (Quince et al., 2017). To address this, we performed targeted culture-based isolation of viable bacteria, followed by whole-genome sequencing of key isolates. This approach allowed direct verification of phenotypic antibiotic and biocide resistance, virulence potential, and mobile genetic element content, while complementing the broader diversity captured by metagenomics. By integrating both approaches, we could accurately resolve both the taxonomic and functional architecture of shaver-associated microbial communities.

Our analyses revealed that shaver micro-niches are overwhelmingly dominated by skin commensals, particularly *Staphylococcus epidermidis* and *Cutibacterium acnes*, but are punctuated by low-abundance opportunistic pathogens, including *Klebsiella pneumoniae*, *Acinetobacter ursingii*, and *Achromobacter insuavis*. These taxa persisted despite the absence of clinical antibiotics, suggesting adaptation to non-clinical selective pressures such as metal alloys and biocidal chemicals. Functional profiling of metagenomes showed enrichment of genes conferring metal tolerance (e.g., *ars*, *cop*, *czc* operons) and biocide resistance (e.g., *fabI*, *smrA*), alongside multidrug resistance determinants, including efflux pumps, target modifications, and rare “last-resort” antibiotic resistance genes. Virulence profiling revealed factors mediating adhesion, biofilm formation, and tissue damage, including autolysins, fimbrial adhesins, and necrotizing toxins. Network analyses further highlighted co-occurrence patterns between resistance and virulence genes and mobile genetic elements such as transposases, integrases, and IS elements, suggesting the potential for horizontal gene transfer.

Culture-based analyses confirmed that these opportunistic pathogens are viable, with progressive accumulation over repeated use. Whole-genome sequencing of 45 representative isolates reconstructed large resistance islands and conserved heavy metal–resistance scaffolds, revealing standardized genetic architectures that co-integrate biocide tolerance, metal resistance, multidrug resistance, and virulence traits. Collectively, these data demonstrate that electric shavers function not only as refuges for potential pathogens but also as selective microenvironments that may facilitate the persistence and horizontal dissemination of multidrug resistance and virulence factors within human-associated microbiomes.

## 2 Materials and methods

### 2.1 Shaver standardization and temporal sampling

A cohort of 10 healthy male university students (aged 18–25 years) was recruited for this study. All participants were screened to ensure they had no history of chronic skin diseases (e.g., atopic dermatitis or psoriasis), had not utilized systemic or topical antibiotics within the past 3 months, and used electric shavers as their primary grooming tool. To establish a sterile microbial baseline, all electric shavers underwent a rigorous decontamination protocol on Day 0. The detachable shaver heads were completely submerged in 75% ethanol for 15 min, while the shaver bodies were thoroughly disinfected using 75% ethanol swabs. Participants were instructed to use only these standardized devices for the duration of the 21-day experiment, maintaining a shaving frequency of once every 2–3 days. Sampling was performed at two distinct stages: (i) Early-stage (Day 2): After the initial 2 days of post-sterilization use, the accumulated whiskers and biomass were harvested for targeted bacterial isolation. (ii) Mature-stage (Day 21): After 3 weeks of cumulative grooming, the total biomass (comprising whiskers and microbial biofilm) was harvested. This 21-day cumulative sample was partitioned for both culture-dependent bacterial isolation and total metagenomic DNA extraction.

### 2.2 Microbial isolation and phenotypic identification

To recover viable shaver-associated microbiota, harvested biomass was resuspended in 1 mL of sterile PBS, vortexed vigorously for 1 min, and pre-enriched in Brain Heart Infusion (BHI) broth at 37 °C for 4 h to resuscitate stressed cells. Enriched cultures were serially diluted and streaked onto diverse solid media, including 5% sheep blood agar (BAP), Mannitol Salt Agar (MSA), MacConkey agar (MAC), and Bile Esculin Agar (BEA). In addition, inoculated BAP plates were incubated under anaerobic conditions at 37 °C for up to 7 days to improve recovery of anaerobic skin-associated taxa. Representative colonies were purified on Tryptic Soy Agar (TSA) and identified to the species level using MALDI-TOF MS (Patel, 2015) (Bruker Daltonics). Notably, despite the high metagenomic abundance of *C. acnes*, no viable *C. s*trains were recovered under the culture conditions used in this study. This discrepancy may reflect reduced viability, methodological limitations of the isolation protocol, including the choice of media and enrichment conditions, or both, and therefore should not be interpreted as direct evidence of an active *Cutibacterium*-associated resistome. Pure cultures were cryopreserved in TSB with 25% glycerol at −80 °C. Air-exposed environmental swabs and reagent blanks were processed simultaneously as negative controls; all remained completely sterile after prolonged incubation, confirming the reliability of our isolation pipeline.

### 2.3 Targeted PCR validation of *mcr* variant and *tet(X)* genes

To validate the presence of the putative *mcr* and *tet*(X) resistance determinants detected by metagenomic read mapping, targeted PCR assays were performed using total DNA extracted from the corresponding shaver biomass samples. Primer sets targeting representative *mcr* family genes and *tet(X)* family sequences were selected from previously published studies and used to amplify conserved regions of the detected resistance gene sequences. PCR amplification was carried out in a 25 μL reaction mixture containing 12.5 μL of 2 × Taq PCR Master Mix, 0.5 μM of each primer, 1–2 μL of template DNA, and nuclease-free water. The amplification program consisted of an initial denaturation at 95 °C for 3 min, followed by 35 cycles of denaturation at 95 °C for 30 s, annealing at 58 °C for 30 s, and extension at 72 °C for 30 s, with a final extension at 72 °C for 5 min. PCR products were resolved by electrophoresis on 1.5% agarose gels and visualized under UV illumination. Amplicons of the expected size were interpreted as positive signals for the corresponding target gene. Because no viable carrier isolates were recovered for these targets, the PCR assays were used to confirm the presence of the corresponding resistance determinants at the community DNA level rather than to assign them to specific cultured strains. Primer names, sequences, and expected amplicon sizes are listed in Supplementary Table S1.

### 2.4 Isolates whole-genome sequencing and bioinformatic characterization

Total genomic DNA were extracted from pure isolates using the PureLink Genomic DNA Minikit (Invitrogen, United States). Whole-genome sequencing (WGS) was exclusively performed on isolates recovered from subjects with paired metagenomic data. Sequencing was conducted on the DNBseq-T7 platform (Jeon et al., 2021) (paired-end, 250 bp). Raw reads were strictly quality-filtered using SOAPnuke (parameters: -n 0.01 -l 20 -g 0.4 --adaMis 3 --outQualSys 1 --minReadLen 150), and *de novo* assembly was executed using SPAdes v3.15, retaining contigs >500 bp (Bankevich et al., 2012). For genomic profiling, open reading frames (ORFs) were annotated via the RAST server and NCBI PGAP. The resistome, virulome, and mobilome were comprehensively screened against ARGs-OAP, CARD, VFDB, and MEG databases (updated Feb 2026) (Bortolaia et al., 2020). structural comparative analyses and the localization of antibiotic resistance genes (ARGs) and mobile genetic elements (MGEs) were visualized with BRIG and Easyfig. Finally, high-resolution phylogenetic reconstruction of the isolates was performed using PhyloPhlAn v3.0 (Segata et al., 2013) and visualized via the iTOL platform (Letunic and Bork, 2024) to elucidate evolutionary relationships.

### 2.5 Metagenomic DNA extraction and sequencing

For metagenomic analysis, total community DNA was recovered from the skin swabs. Each swab was submerged in 1 mL of sterile PBS and subjected to high-speed vortexing for 2 min to ensure the maximum release of microbial biomass. The resulting suspension was processed using the QIAamp DNA Microbiome Kit, following the manufacturer’s instructions, which included a mechanical lysis step to ensure the extraction of DNA from Gram-positive organisms. Library construction was initiated by enzymatically shearing the purified genomic DNA into fragments with a target size of 350 bp. These fragments underwent end-repair, A-tailing, and ligation with sequencing-specific adapters, followed by a low-cycle PCR enrichment. To prepare the libraries for the DNBseq platform, the double-stranded PCR products were thermally denatured and covalently closed using DNA ligase to form single-stranded circular DNA (ssCir DNA) templates. Any remaining linear DNA was removed via exonuclease treatment. The integrity and concentration of the resulting circular libraries were validated using Qubit fluorometric quantification, qPCR, and an Agilent Bioanalyzer to assess size distribution. Qualified libraries were transformed into DNA nanoballs (DNBs) through rolling circle replication and sequenced on the DNBseq-T7 platform in a paired-end 150 bp (PE150) configuration, yielding approximately 20 Gb of raw data per sample. Meanwhile, the DNA concentrations of all environmental negative controls were below the limit of detection via Qubit fluorometric quantification, and subsequent attempts at library construction resulted in failure due to negligible DNA yields, thereby confirming the sterility of the sampling and experimental procedures.

### 2.6 Metagenomic sequencing and bioinformatic profiling

**Figure 1:**
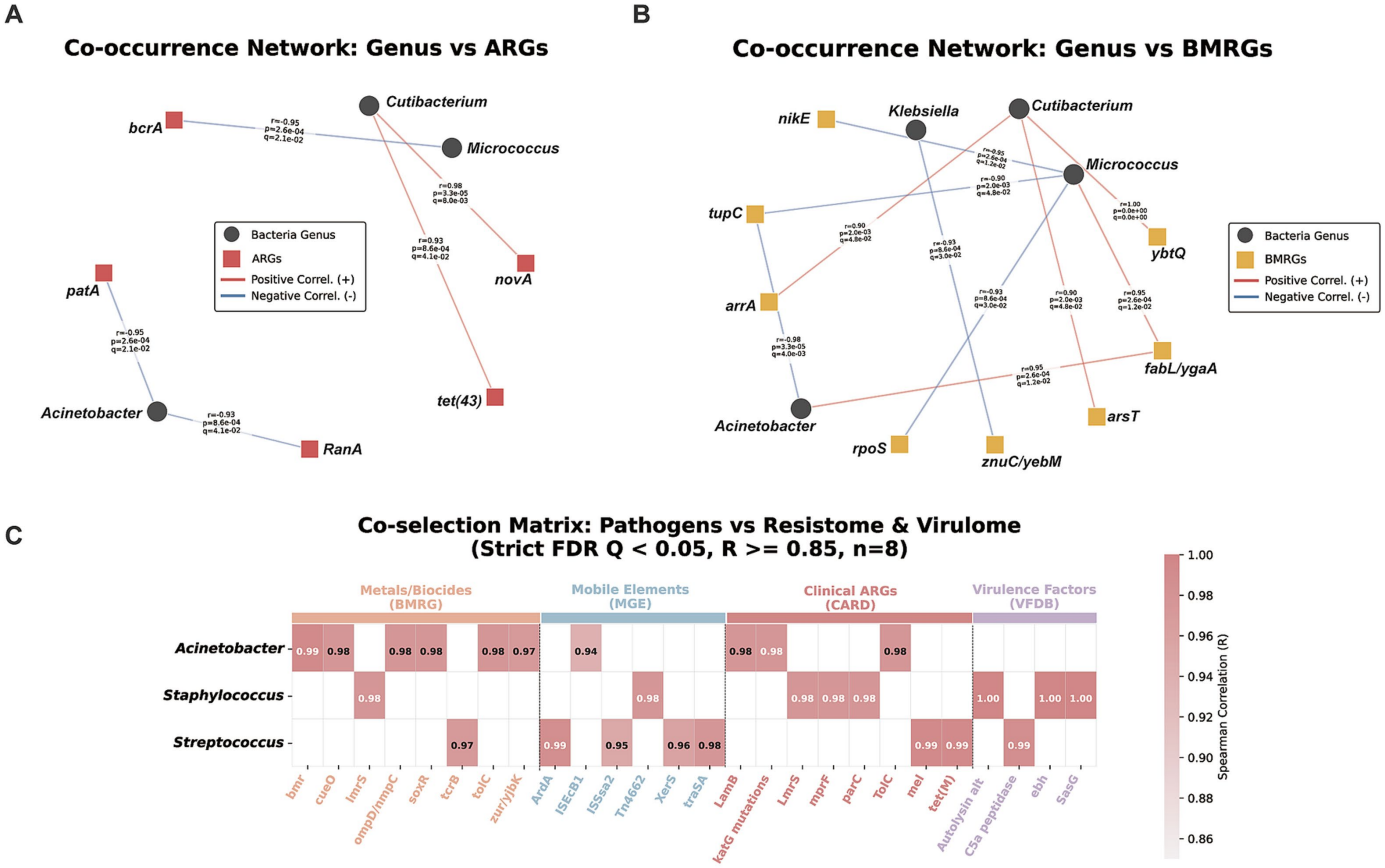
Co-occurrence networks and pathobiome co-selection matrix for target pathogens. **(A,B)** Co-occurrence networks showing significant associations between core potential pathogens and **(A)** metal/biocide resistance genes (BMRGs) and **(B)** clinical antibiotic resistance genes (ARGs). Nodes represent bacterial genera or functional genes, and edges indicate robust correlations (Spearman’s |ρ| ≥ 0.85). **(C)** Integrated pathobiome co-selection matrix across four functional pillars: BMRG, mobile genetic elements (MGE), clinical ARGs (CARD), and virulence factors (VFDB). The heatmap displays top representative functional modules positively correlated with target pathogens (Spearman’s |ρ| ≥ 0.80). To robustly mitigate the risk of spurious correlations associated with a limited sample size (*n* = 8), all displayed interactions were strictly filtered. Only correlations satisfying an exploratory significance threshold of *p* < 0.01 or a Benjamini–Hochberg False Discovery Rate (FDR) corrected *Q* < 0.05 were retained for visualization. Features present in fewer than three samples were excluded to ensure ecological relevance. For example, *Cutibacterium* showed a perfect correlation with the iron-scavenging system *ybtQ* (*R* = 1.0, *Q* < 0.001) and the clinical ARG *novA* (*R* = 0.98, *Q* < 0.01). A comprehensive list of all significant co-occurrence pairs is provided in Supplementary Table S2.

For metagenomic analysis, total community DNA was extracted from the Day 21 cumulative shaver biomass. Each harvested sample was resuspended in 1 mL of sterile PBS, vortexed vigorously for 2 min, and processed using the QIAamp DNA Microbiome Kit, which incorporates mechanical lysis to ensure the robust recovery of Gram-positive organisms. Sequencing libraries with a target insert size of 350 bp were constructed and sequenced on the DNBseq-T7 platform utilizing a paired-end 150 bp strategy, yielding approximately 20 Gb of raw data per sample. Environmental negative controls remained below the detection limit, confirming the absolute sterility of the sampling pipeline. Following sequencing, raw metagenomic reads underwent rigorous quality control via Trimmomatic (v0.39) to eliminate adapters and low-quality bases (<Q15), while host-derived human DNA contamination was effectively depleted using KneadData (v0.7.7). The resulting high-quality clean reads from the 8 successfully sequenced shaver metagenomes were assembled *de novo* into contigs using MEGAHIT (v1.2.9; Li et al., 2015), with structural integrity (e.g., N50 metrics) validated via MetaQUAST (v5.0.2) (Mikheenko et al., 2016). Taxonomic composition was profiled using MetaPhlAn4 against the CHOCOPhlAnSGB_202503 database. To further resolve low-abundance taxa within the rare biosphere, predicted protein-coding genes from the assemblies were aligned against the NCBI Non-Redundant (NR) database, and identified species were cross-referenced with the gcPathogen database. Community structural complexity was evaluated through Alpha and Beta diversity indices (e.g., Principal Coordinate Analysis based on Bray–Curtis dissimilarity). To elucidate the metabolic mechanisms underlying bacterial survival in the extreme shaver micro-niche, functional annotation was conducted by mapping predicted genes to the KEGG database. For the comprehensive profiling of the resistome, virulome, and mobilome, sequences were aligned against specialized databases, including CARD (for ARGs), VFDB (for virulence factor genes, VFGs), BacMet (Pal et al., 2014) (for biocide and metal resistance genes, BMRGs), and specific MGE databases. The quantitative profiles of these functional determinants (ARGs, VFGs, BMRGs, and MGEs) were determined by calculating the total mapped read counts for each assigned gene. These raw counts were subsequently normalized to compute the relative abundances of the functional traits across the metagenomic communities. Given the limited number of metagenomic samples (*n* = 8), Spearman’s rank correlation analysis was treated as exploratory. To reduce the risk of spurious associations arising from multiple comparisons, *p* values were adjusted using the Benjamini–Hochberg false discovery rate (FDR) procedure, and associations with *q* < 0.05 were considered statistically significant. These exploratory correlations were used to describe potential co-occurrence trends and were interpreted cautiously in light of the small sample size. We further analyzed the co-occurrence between core bacterial taxa and four functional databases (BMRG, CARD, VFDB, and MGE). To ensure data reliability, only taxa and genes present in at least three of the eight samples were included. For the co-occurrence networks (Figures 1A,B), only robust associations with Spearman’s |ρ| ≥ 0.85 and an exploratory significance threshold of *p* < 0.01 or FDR-corrected *q* < 0.05 were retained. For the integrated pathobiome co-selection matrix (Figure 1C), the correlation threshold was set at ρ ≥ 0.80 under the same statistical criteria. To improve interpretability and visual clarity, up to five top-correlated representative genes per functional category were displayed for each pathogen. Network visualization was performed using Gephi, and the pathobiome matrix was generated using the seaborn library in Python (Supplementary Table S2).

## 3 Results

### 3.1 The electric shaver as an anthropogenic niche dominated by skin commensals and punctuated by opportunistic pathogens

**Figure 2:**
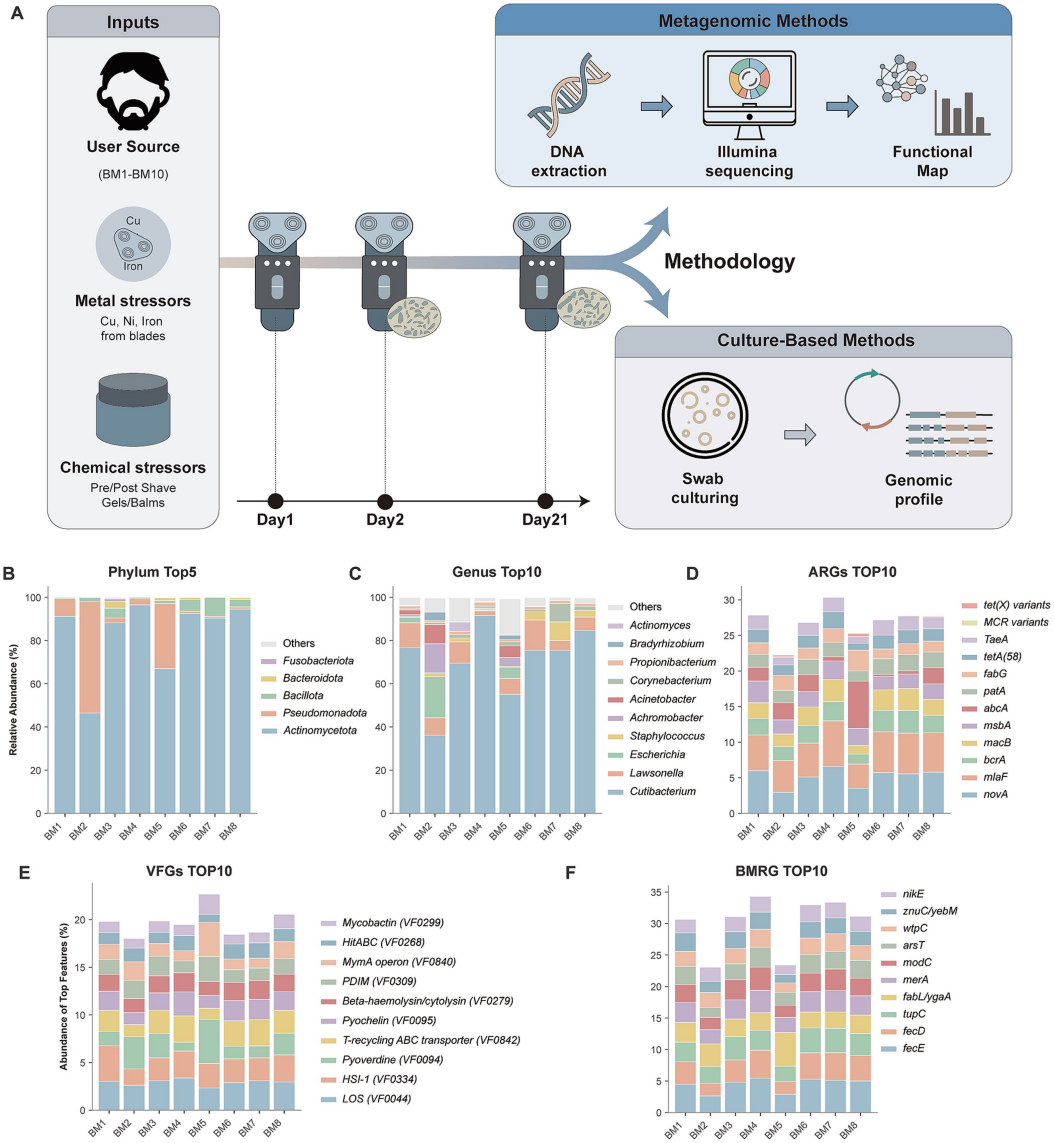
Experimental design and the taxonomic and functional landscape of the electric shaver microbiome. **(A)** Schematic overview of the study design, illustrating the standardized decontamination, temporal sampling (Day 2 and Day 21 cumulative biomass), and the integrated pipeline of deep metagenomic sequencing and culture-dependent targeted isolation across 10 healthy individuals. **(B,C)** Taxonomic composition of the shaver micro-niche, displaying the relative abundance of the top 5 most abundant microbial phyla **(B)** and the top 10 bacterial genera **(C)** across the metagenomic samples. **(D,F)** Comprehensive functional profiling of the shaver resistome, virulome, and mobilome. The relative abundances for all functional determinants were calculated based on the percentage of total mapped read counts. **(D)** The relative abundance of the top 10 antibiotic resistance genes (ARGs), with a specific highlight on the detection of clinical “last-resort” resistance determinants (*mcr* and *tet*(X) variants) within the microbial community. **(E)** The profile of the top 10 virulence factor genes (VFGs). **(F)** The relative abundance of the top 10 biocide and metal resistance genes (BMRGs), highlighting the fundamental genetic repertoire driving bacterial survival under extreme anthropogenic physicochemical stress.

To elucidate the microbial architecture of the shaver micro-niche, we conducted deep metagenomic sequencing on samples collected from a cohort of 10 healthy individuals (seven utilizing biocide-containing shaving foam and three without) after 21 days of continuous use. Owing to the ultra-low biomass inherent to the initial stages of colonization (Day 2), our metagenomic profiling focused on the mature communities successfully recovered from eight representative samples (BM1–BM8) (Figure 2A). The taxonomic landscape was overwhelmingly defined by skin-associated features, reflecting a high-frequency biological exchange between the host epidermis and the device. At the phylum level, the communities were predominantly assembled by *Actinomycetota* (average relative abundance 83.4%), followed by *Pseudomonadota* (12.2%) and *Bacillota* (3.3%). This skin-seeded baseline was further corroborated at the genus level, where *Cutibacterium* maintained a stark dominance (70.6%), alongside *Lawsonella* (7.9%) and *Corynebacterium* (2.1%) (Figure 2B; Supplementary Table S3). Collectively, these profiles establish the shaver as a robust *ex vivo* sanctuary for human skin commensals.

Beneath this commensal-dominated baseline, however, the physicochemical constraints of the shaver appeared to drive distinct community succession and the localized expansion of atypical taxa. Notably, samples BM2 and BM5 exhibited a dramatic structural shift, marked by an abnormal surge in *Pseudomonadota* (reaching 51.5 and 30.1%, respectively). Within these functionally perturbed micro-environments, we detected an enriched presence of Gram-negative genera classically recognized as potent vectors for multidrug resistance (MDR), including *Escherichia* (3.4%), *Achromobacter* (2.4%), and *Acinetobacter* (2.1%) (Figure 2C). Samples BM2, BM3and BM5 were obtained from individuals who did not use biocide-containing foam, and ordination analysis suggested partial separation of these samples from the remaining cohort (Supplementary Figure S1). To statistically validate the community separation driven by shaving foam usage, a Permutational Multivariate Analysis of Variance (PERMANOVA) was performed. The multivariate analysis confirmed that foam usage significantly altered the microbial community structure (PERMANOVA, Pseudo-*F* = 4.59, *p* = 0.036), corroborating the distinct clustering observed in the PCA. Although this pattern is suggestive, it should be interpreted cautiously because several potentially relevant variables—including shaver brand, blade alloy, and user hygiene practices—were not standardized in the present study. High-resolution species-level profiling further illuminated the covert establishment of clinically relevant opportunistic pathogens within the “rare biosphere” of this niche. While *C. acnes* remained the dominant taxon in the metagenomic profiles, its high DNA abundance should not be interpreted as evidence of viability or metabolic activity in the absence of dedicated viability assessment.

### 3.2 Functional convergence and dynamic mobilization of the resistome and virulome

To contextualize the resistome, global functional profiling revealed a community under severe physical and nutritional duress. The baseline metabolic framework (top 10 MetaCyc pathways) (Supplementary Figure S2; Supplementary Table S9) was highly enriched in peptidoglycan biosynthesis and the chorismate superpathway, reflecting crucial adaptations for cell wall maintenance against blade friction and trace metal scavenging. Concurrently, the most abundant KEGG orthologs (KOs) were overwhelmingly driven by stress-response and transmembrane transport systems (Supplementary Figure S3; Supplementary Table S8). The absolute dominance of MFS and ABC transporters, alongside critical iron-complex transport (K02016) and DNA repair helicases (K03657), established a transporter-heavy physiological foundation. Given this architecture, the micro-environment exerted a profound physicochemical selective regime, primarily imprinted on the biocide and metal resistance gene (BMRG) profiles. The BMRG reservoir was robustly defined by its top three classes: pure metal tolerance (65.57%), biocide resistance (24.36%), and dual-resistance mechanisms (10.07%) (Figure 2D; Supplementary Table S4). Specifically, we detected a high enrichment of *copR*, *copA*, and *czcD* against metal exudation, alongside *fabI* and *smrA* conferring robust tolerance to shaving foam chemicals. Strikingly, this non-clinical selective pressure was coupled with a formidable antibiotic resistome. Utilizing the highly enriched transport systems, the communities predominantly deployed the top three resistance mechanisms—antibiotic efflux pumps (47.74%) (Figure 2E; Supplementary Table S5), target alterations (36.48%), and target protection (5.95%)—to counteract the top three most enriched drug classes in this niche: peptide (11.79%), tetracycline (8.21%), and glycopeptide (6.02%) antibiotics. Embedded within this broad-spectrum baseline, we detected metagenomic signatures assigned to several clinically important resistance gene families, including van clusters (*vanR/K/B*), methicillin-resistance-associated markers (*mecA/mecC*), and putative plasmid-mediated colistin (*mcr*) and tigecycline *[tet(X)]* resistance genes. Targeted PCR further confirmed the presence of *mcr-*variant and *tet*(X) sequences in the corresponding samples. However, because no viable carrier isolates were recovered and no phenotypic validation was performed, these genes were not considered functionally confirmed in specific bacterial strains in the present study.

Beyond merely surviving the physicochemical constraints, the colonizing taxa maintained a sophisticated virulome and a highly dynamic mobilome. To adapt and potentially transition to pathogenesis, the virulence factor (VF) profile was primarily optimized around its top three categories: nutritional and metabolic factors for resource scavenging in a depleted environment (33.62%), immune modulation for host evasion (20.06%), and effector delivery systems (9.81%) (Figure 2F; Supplementary Table S6). Crucially, within this mainstream arsenal, we unambiguously identified severe necrotizing toxins, such as Panton-Valentine leukocidin (PVL) and alpha-hemolysin (*hla*). In the context of micro-abrasions routinely inflicted during shaving, the presence of these tissue-damaging toxins highlights the potential for transitioning from benign colonization to skin and soft tissue infections (SSTIs). Underpinning the assembly and dissemination of these resistance and virulence traits is a hyperactive mobilome. The niche is characterized by an extraordinary frequency of horizontal gene transfer (HGT) machinery, overwhelmingly defined by its top three mobile elements: transposases (37.99%), integrases (5.52%), and IS630 insertion sequences (4.83%) (Supplementary Table S7). The ubiquitous presence of the bla-disseminating Tn3 family (1.23%) and the detection of the clinical class 1 integron marker (*IntI1*) suggest that this micro-niche may serve as a potential setting for the ongoing reshuffling and horizontal mobilization of multidrug resistance traits.

### 3.3 Co-occurrence networks delineate the integration of environmental tolerance, multidrug resistance, and clinical virulence

To explore potential associations between the physicochemical environment of the shaver micro-niche and the distribution of functional genetic modules, we constructed high-resolution co-occurrence networks and a multi-dimensional “four-pillar” pathobiome matrix (integrating BMRG, CARD, VFDB, and MGE) (Figure 1). Given the limited sample size (*n* = 8) and the exploratory nature of this analysis, we implemented a rigorous statistical framework to mitigate the risk of spurious correlations. Only robust associations—defined by Spearman’s |*ρ*| ≥ 0.85 for networks and ρ ≥ 0.80 for the matrix—that passed Benjamini-Hochberg False Discovery Rate (FDR) correction (*q* < 0.05) or strict exploratory thresholds (*p* < 0.01) were interpreted. While these patterns offer critical ecological insights into co-selection, they are presented as hypothesis-generating evidence of functional linkage rather than definitive biological proof of co-localization.

As the dominant colonizer, *Staphylococcus* exhibited a sophisticated functional architecture intertwining stress tolerance with clinical risk. Its sustained dominance was deeply interlocked with an arsenal of BMRGs targeting biocides and metals, notably the multidrug efflux system *ykkC/ykkD* and *lmrS* (Figure 1B). This physicochemical defense was statistically coupled with lethal mutation-driven clinical ARGs, including *mprF* (daptomycin resistance) and the multidrug transporter *norB*, alongside a suite of tissue-adhesion and biofilm-forming virulence factors such as autolysin *alt*, clumping factor A (*clfA*), and the giant protein *ebh*. This genetic payload showed strong positive correlations with site-specific recombinases and transposases, suggesting that *Staphylococcus* may leverage mobile genetic vehicles to maintain its niche dominance and clinical invasiveness.

Beyond the staphylococcal dominance, opportunistic pathogens employed divergent strategies to couple resistance and virulence. *Acinetobacter*, a notorious clinical opportunist, displayed the most extensive and complex functional linkages in our cohort. Its survival uniquely coupled environmental stress sensors (e.g., tungsten transport *tupC* and efflux *emrA*) with critical clinical ARGs, including mutations in *gyrA* and *parC* (fluoroquinolone resistance) and *katG* (isoniazid resistance) (Supplementary Table S2). These traits were highly correlated with a vast array of insertion sequences (IS elements, e.g., IS*3*-family) and transposases (ρ > 0.95, *q* < 0.001), highlighting the pivotal role of IS-mediated mobilization in capturing and assembling foreign resistance traits within *Acinetobacter* populations.

Conversely, *Streptococcus* adopted a strategy heavily biased toward virulence and specific antibiotic protection. Its abundance was tightly bound to a myriad of virulence factors, particularly tissue-adhesion pili (e.g., SpaA-type) and evasion proteins (e.g., *SphB1*), alongside clinical ARGs like *tet(M)* (ribosomal protection) and *nreB*. These associations precisely matched the high-abundance signals of conjugative transposons, indicating a propensity for mobilizing these traits as massive, conjugated functional blocks.

Within the lower-abundance “rare biosphere,” pathogens like *Cutibacterium*, *Klebsiella*, and *Pseudomonas* exhibited specialized survival signatures. *Cutibacterium* showed a strong positive correlation with the iron-scavenging yersiniabactin transport system *ybtQ* (ρ = 1.0, *q* = 0.0) and the clinical ARG *novA* (ρ = 0.98, *q* < 0.01), suggesting a possible association between resource scavenging and specific antibiotic defense. *Klebsiella* was associated with high-affinity siderophore systems for iron acquisition together with the clinical resistance gene *vanG* and Type III secretion system (T3SS) components. Similarly, *Pseudomonas* was associated with tellurite tolerance (*klaB/telA*) and fluoroquinolone efflux (*norC*). Together, these taxa-specific patterns are consistent with the possibility that the shaver micro-environment may favor the co-occurrence of environmental stress tolerance, clinical virulence, and multidrug resistance.

### 3.4 Targeted isolation of viable opportunistic pathogens reveals temporal accumulation and overcomes metagenomic blind spots

**Figure 3:**
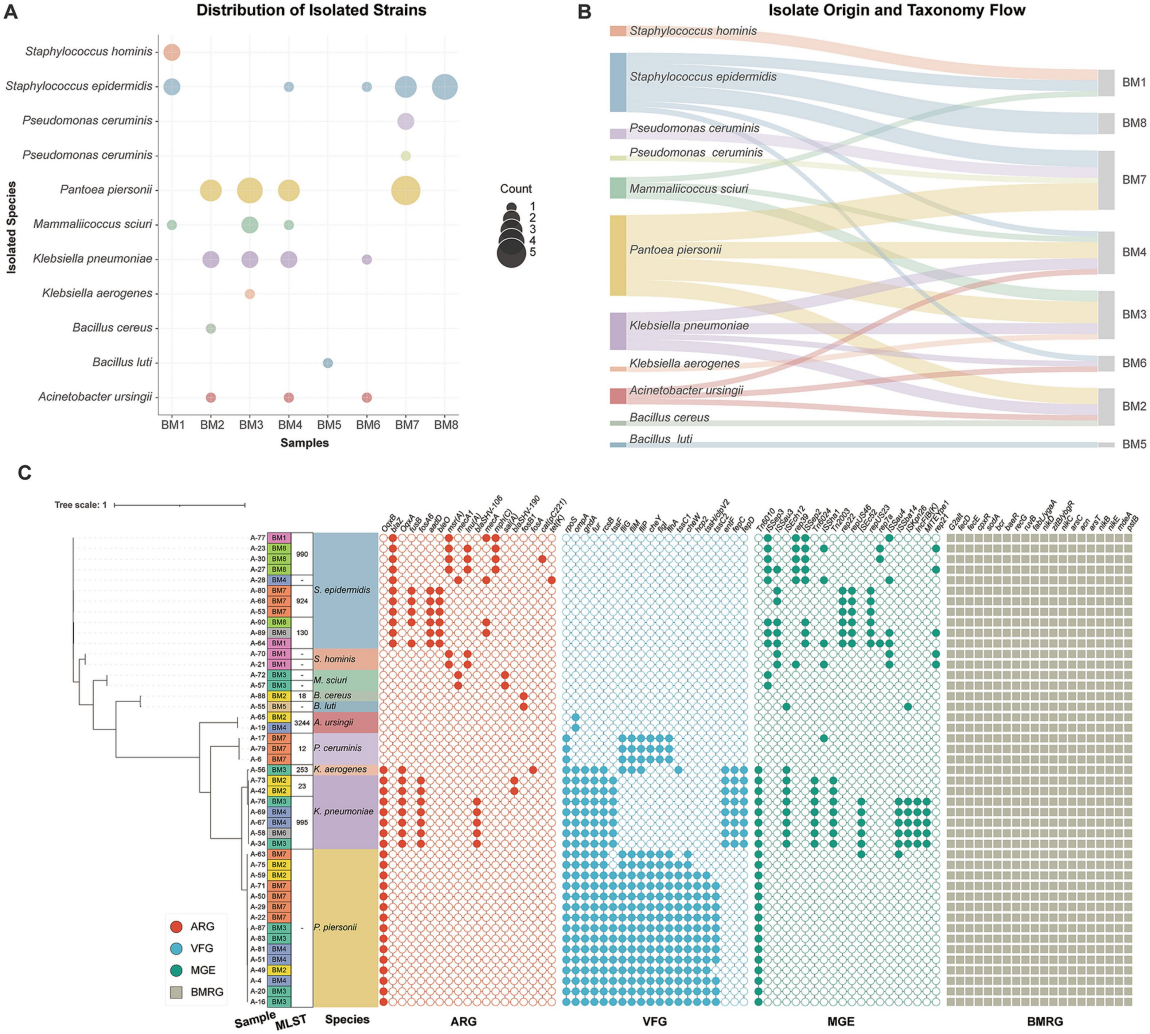
Targeted isolation, source tracking, and phylogenomic profiling of the viable shaver microbiota. **(A)** Bubble chart illustrating the quantitative recovery profile of viable bacterial isolates. Bubble sizes correspond to the number of strains successfully cultivated, highlighting the taxonomic diversity and isolation yield across different samples. **(B)** Sankey diagram mapping the source distribution of the recovered microbiota, tracing the specific connections between the isolated bacterial taxa and their respective human shaver origins (e.g., BM2, BM3, etc.). **(C)** High-resolution core-genome phylogenetic tree of the sequenced viable isolates. Concentric rings surrounding the dendrogram denote specific strain metadata, including species identity, sample origin, and multilocus sequence typing (MLST) assignment (revealing clonal convergence, such as ST23, ST995, and ST3244). The outer heatmaps detail the genotypic carriage of mobile genetic elements (MGEs), clinical antibiotic resistance genes (ARGs), and virulence factor genes (VFGs). Notably, for biocide and metal resistance genes (BMRGs), the visualization explicitly highlights a highly conserved core subset of genes universally carried by all sequenced isolates, representing the fundamental genetic prerequisites for survival in this extreme anthropogenic micro-niche.

While metagenomics illuminated the vast resistance potential within the shaver micro-niche, it inherently conflates viable colonizers with extracellular DNA relics and often lacks the resolution to pinpoint specific agents within the “rare biosphere.” To definitively assess the actual transmission risk of living pathogens, we performed targeted, culture-dependent isolation across ten independent shaver samples (BM1–BM10) with varying usage durations. We successfully recovered 97 viable, stress-tolerant bacterial strains. Strikingly, the recovery rate exhibited a profound temporal cumulative effect: only 17 strains were isolated from early-stage samples (Day 2), whereas the vast majority (80 strains) were robustly established in mature samples (Day 21) (Figures 3A,B; Supplementary Table S10). This quantitative disparity directly captures the progressive colonization and persistent expansion of surviving taxa under continuous physicochemical stress. Furthermore, the isolation yield was highly concentrated in specific samples—namely BM4 (20 strains), BM3 (16), and BM2 (14)—perfectly mirroring the functional “hotspots” of ARGs and BMRGs previously identified in our metagenomic profiling, thereby functionally linking community biomass to resistome density.

Beyond quantitative validation, the culture-dependent approach provided crucial taxonomic ground-truth, unambiguously proving that the high-risk opportunists detected *in silico* are indeed living colonizers. Utilizing MALDI-TOF MS for initial high-throughput screening, coupled with high-quality whole-genome sequencing (WGS) of 45 meticulously selected core strains, we precisely resolved the taxonomy of the 97 isolates into 16 distinct viable species (Figure 3C). Crucially, this viable cohort perfectly recapitulated the core metagenomic signatures. We successfully revived both the dominant skin commensals (e.g., *Staphylococcus epidermidis* and *S. capitis*) and, more alarmingly, the extremely low-abundance but high-risk clinical superbugs, including *K. pneumoniae*, *Klebsiella aerogenes*, and *Acinetobacter ursingii*. The recovery of these viable opportunists definitively refutes the possibility that their metagenomic signals were mere background DNA noise. Although metagenomics indicated a high abundance of *C. acnes*, no viable isolates were recovered in the present study. Accordingly, these signals are more appropriately interpreted as DNA-based evidence of prior or residual microbial presence, rather than proof of an active resistome associated with viable *Cutibacterium* cells. Moreover, the targeted isolation successfully bypassed the resolution limits of short-read metagenomics by uncovering a “hidden” reservoir of 10 additional species (such as *Staphylococcus hominis*, *Mammaliicoccus sciuri*, *Pantoea piersonii*, and *Pseudomonas ceruminis*). These viable taxa, likely obscured as ambiguous genus-level clusters in the meta-data, highlight the indispensable role of pure cultivation in capturing the complete functional landscape of the rare biosphere.

Strikingly, multilocus sequence typing (MLST) and whole-genome phylogenetic analysis of the sequenced cohort revealed a recurrent lineage concentration within the shaver micro-niche. Rather than displaying the high genetic diversity typical of random environmental contamination, opportunistic pathogens recovered from physically isolated shavers exhibited marked lineage concentration. For instance, the recovered *K. pneumoniae* strains from different individuals (e.g., BM2, BM3, BM4, BM6) were confined to only two sequence types (MLST995 and MLST23). Similarly, all *Acinetobacter ursingii* isolates belonged to a single lineage (MLST3244), while the dominant commensal *Staphylococcus epidermidis* was concentrated in specific types such as MLST130 and MLST990. Crucially, core-genome phylogenetic reconstructions indicated that while these cross-sample isolates were phylogenetically related, they were not identical clones originating from a single contamination event. This population structure is consistent with recurrent selection and enrichment of specific stress-tolerant lineages across different human hosts.

### 3.5 Recurrent genomic architectures and shared MLST profiles suggest repeated selection of related resistance-associated lineages in the shaver micro-niche

To transcend the limitations of single-strain analysis and globally evaluate the structural universality of co-selection, we constructed a “Total Contig Matrix” encompassing thousands of multi-resistance contigs extracted from both the WGS isolates and the overall metagenome-assembled data. Upon scanning this global matrix, a fundamental prerequisite for survival immediately emerged: despite extreme taxonomic divergence, the viable isolates exhibited a striking universal carriage of a core biocide and metal resistance gene (BMRG) repertoire (ubiquitously harboring the *ars* and *nik* operons, *baeR*, and *fabL/ygaA*). This ubiquitous BMRG baseline underscores that basal heavy metal tolerance acts as the absolute gatekeeper for colonization (Figure 3C).

Building upon this shared survival foundation, the matrix revealed an astonishing network of genetic recombination. Under relentless environmental pressure, bacteria tend to cluster functional genes into massive arrays. We identified widespread mega-islands (>100 kb) frequently assembled in opportunistic pathogens like *K. pneumoniae* and *K. aerogenes*. Notably, these high-risk taxa explicitly weaponized these mega-islands by co-integrating severe clinical ARGs—such as extended-spectrum beta-lactamases (*bla*_SHV-106_), fluoroquinolone efflux pumps (*oqxA/B*), and fosfomycin resistance determinants (*fosA*)—alongside a formidable arsenal of virulence factors. The concurrent carriage of robust tissue-adherence fimbriae (*mrk* and *fim* operons), high-affinity siderophore systems (*ent* and *fep* operons for iron scavenging), and Type VI secretion systems (T6SS) highlights their immense pathogenic potential to inflict severe infections once the skin barrier is breached by blade micro-abrasions.

**Figure 4:**
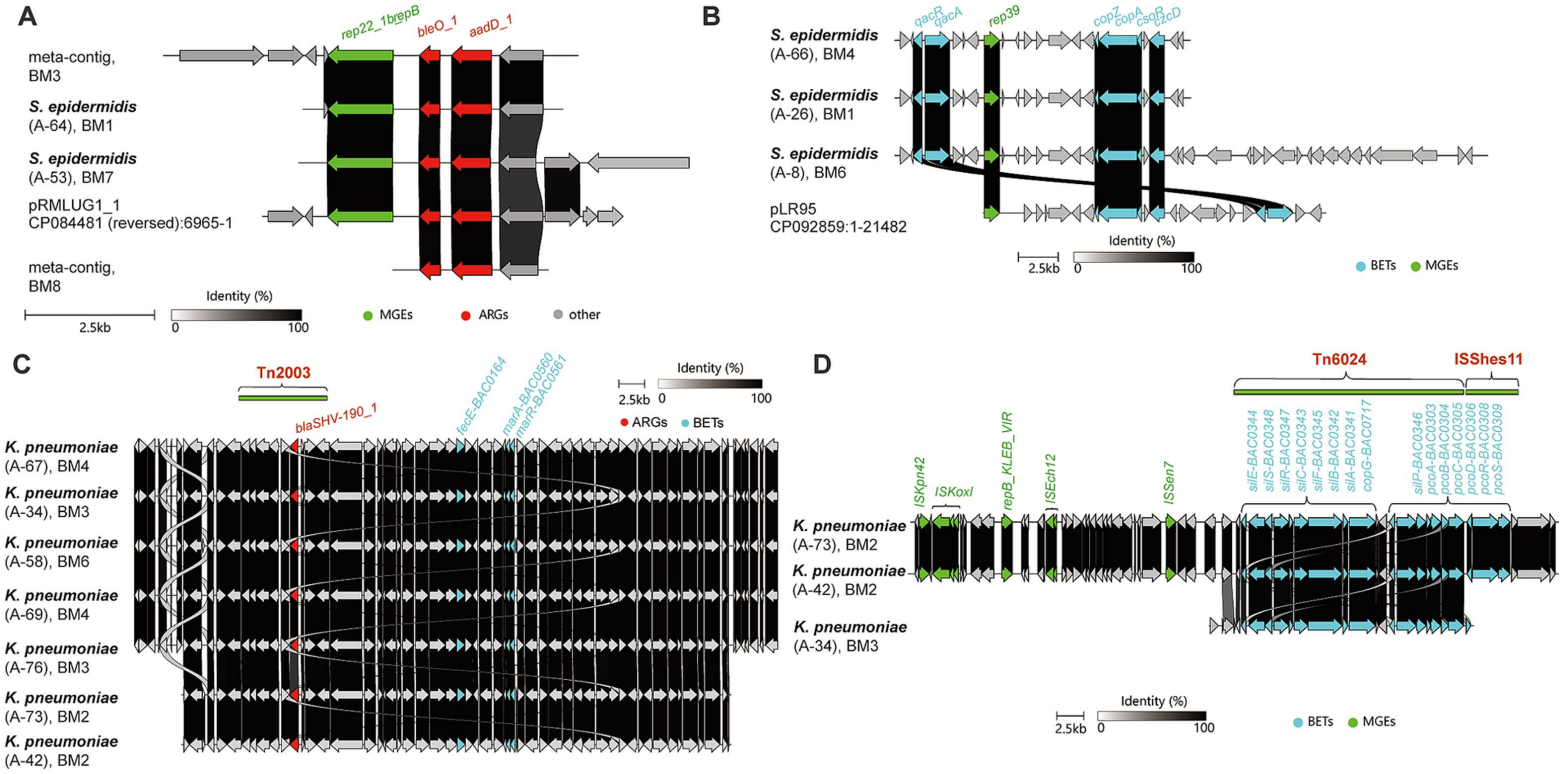
Structural alignments of multidrug resistance cassettes and plasmid backbones revealing physical co-selection. **(A–D)** Linear genomic synteny and structural alignments of representative multidrug resistance (MDR) islands and mobile genetic elements (MGEs) recovered from the viable opportunistic pathogens. The alignments visually confirm the exact physical linkages among biocide/metal resistance genes (BMRGs), clinical antibiotic resistance genes (ARGs), and MGEs. **(A,B)** Fine-scale structural comparisons of resistance cassettes in *Staphylococcus epidermidis*. Panel A highlights the direct physical linkage between specific clinical resistance determinants (*bleO*, *aadD*) and a plasmid replication initiator, *rep22_1b_repB* (pAMα1), underscoring their mobilization potential. Panel B illustrates the clustering of the biocide efflux system (*qacA/R*) and heavy metal resistance operons (e.g., *copA/Z* for copper and *czcD*) flanking the MGE *rep39_1_repA* (SAP110A). **(C,D)** Structural alignments of chromosomal and plasmid-mediated mega-resistance regions in *K. pneumoniae*. Panel C demonstrates a classic co-selection architecture where the ESBL determinant *bla*_SHV-106_ (ARG) is mobilized by the transposon Tn*2003* (MGE) and physically co-integrated with an extensive array of BMRGs (e.g., the *marA/R* multiple stress response regulators and *nikE*). Panel D zooms into the massive, highly mosaic plasmid backbones containing virulence-associated replication elements (e.g., *repB_KLEB_VIR*). These regions exhibit near-perfect syntenic conservation of massive heavy metal resistance operons—including the *sil* (silver), *pco* (copper), and *pbr* (lead) clusters—heavily interspersed with active insertion sequences (e.g., IS*Kpn42*, IS*Kox1*) and the transposon Tn*6024*. This highly conserved physical bundling across independent subjects unequivocally validates the convergent evolution driven by the extreme physicochemical pressures of the shaver micro-niche.

Most strikingly, similar genomic architectures frequently recurred across physically isolated shaver samples. Crucially, multilocus sequence typing (MLST) revealed that this widespread structural conservation is not merely a byproduct of recent clonal spread. Instead, isolates from independent human hosts showed identical MLST profiles (such as MLST130 in *S. epidermidis* and MLST995 in *K. pneumoniae*) alongside these recurring genomic structures. To further evaluate the relatedness of these isolates, we calculated the pairwise average nucleotide identity (ANI) and mapped the reads against a reference genome. All *K. pneumoniae* ST995 isolates shared high genomic similarity, with ANI values exceeding 99.98%. However, high-resolution mapping revealed distinct genetic divergence: core-genome variants ranged from 10 to 306 single nucleotide polymorphisms (SNPs), and accessory genome variations were evidenced by unaligned sequences of 2.5–26.0 kb (Supplementary Tables S11, S12; Supplementary Figure S4). These combined genomic metrics—high ANI coupled with clear SNP and structural variations—confirm that these are genetically distinct isolates. This rules out a single recent cross-contamination event and suggests that the shared mega-islands are stable genetic features maintained across independent samples. Together, these observations suggest that the physicochemical constraints of the shaver may repeatedly favor the persistence of related resistance-associated configurations across different hosts. From this vast matrix, we delineated four of the most prevalent and clinically critical plasmid/island backbones driving multidrug resistance (Figures 4A–D). Panel A (Widespread staphylococcal small plasmids) illustrates a perfectly conserved structure characterized by the tandem arrangement of *bleO* (bleomycin) and *aadD* (aminoglycosides) adjacent to a *rep22* replicon. This identical architecture was captured simultaneously across independent samples within *S. epidermidis* isolates sharing the MLST 130 profile, establishing it as a ubiquitous “survival package.” Panel B (Conserved biocide-metal dual-tolerance backbone) highlights a highly conserved plasmid shared widely among *S. epidermidis*. Driven by a *rep39* replicon, it seamlessly links the classic *qacR-qacA* module (conferring resistance to QACs in shaving foam) with *copZ-copA* and *czcD* (combating copper/zinc exudation from blades). Moving to high-risk opportunists, Panel C (ESBL-biocide co-selection in *K. pneumoniae*) uncovers a severe clinical threat. Here, the Tn*2003* transposon directly anchors the ESBL gene *bla*_SHV-106_ immediately upstream of a massive environmental resistance operon (comprising *marA/R*, the iron transporter *fecE*, and biocide targets *fabL/ygaA* and *fabK*). This ingenious genetic linkage ensures that routine exposure to grooming biocides persistently co-selects and retains this potent ESBL superbug. Finally, Panel D (MGE-driven giant heavy-metal carriers) captures a mega-island in *Klebsiella* (predominantly within the MLST 995 profile) driven by an array of mobile elements (IS*Kpn42*, IS*Ech12*, and Tn*6024*). It concatenates three major heavy metal efflux operons—*sil* (silver), pco (copper), and *pbr* (lead)—into a heavy-duty defense system against mixed alloy toxicity. The highly active IS elements flanking these mega-islands act as genomic “magnets,” dynamically primed to capture and integrate passing clinical ARGs into this unsinkable heavy-metal resistance platform.

## 4 Discussion

This study fundamentally reshapes our understanding of daily anthropogenic fomites in antimicrobial resistance (AMR) dissemination. Rather than acting as passive microbial carriers, our integrated analysis suggests that electric shavers—despite lacking clinical antibiotic exposure—may function as selective microenvironments that favor the persistence of opportunistic bacteria and resistance-associated genetic traits. The dual physicochemical stress from blade alloy heavy metals and residual grooming biocides drives intense directional selection, reshaping the skin microbiota and providing a privileged sanctuary for opportunistic pathogens to integrate multidrug resistance and virulence traits. Importantly, the apparent association between foam use and community composition should not be overinterpreted. Because several potentially relevant variables—including shaver brand, blade alloy, and user hygiene practices—were not standardized, the present dataset does not allow firm attribution of the observed separation exclusively to foam use. Nevertheless, the observed pattern is consistent with the possibility that disinfecting foam may help limit the persistence or expansion of opportunistic bacteria on electric shavers, suggesting a potentially relevant preventive role in reducing microbial risk during routine personal care. This potential relevance is supported by recent clinical evidence linking contaminated shaving razors to *Klebsiella*-associated nosocomial transmission (Jiang et al., 2024), as well as by experimental work showing that stainless steel surfaces can support the persistence of pathogenic bacteria and the horizontal transfer of antibiotic resistance determinants (Warnes et al., 2012). These extreme constraints constitute a rigorous ecological filter. On nutrient-depleted, toxic alloy surfaces, successfully established viable strains uniformly carried core biocide and metal resistance genes (BMRGs), such as *ars*, *nik*, and *baeR*, as apparent survival-associated features. Crucially, the high-resolution MLST profiling of viable isolates supports a pattern of lineage enrichment rather than direct evidence of *de novo* evolution. Rather than displaying random genetic diversity, pathogens recovered from physically isolated shavers showed marked lineage concentration: *K. pneumoniae* isolates were largely restricted to the MLST995 and MLST23 profiles, while all *Acinetobacter ursingii* isolates belonged to MLST3244. Genomic profiling provided additional evidence for this environmental filtering. While the *K. pneumoniae* isolates were closely related (ANI > 99.98%), they exhibited distinct genetic markers, including core-genome SNPs (10–306) and substantial accessory genome structural variations (up to 26.0 kb of unaligned sequences) (Supplementary Tables S11, S12; Supplementary Figure S4). These differences confirm that the isolates represent distinct colonization events rather than artifacts of sampling contamination. The observation that these genetically distinct isolates consistently maintained identical mega-islands suggests a strong selection pressure within the shaver environment that favors the stability and retention of these specific resistance modules. Because core-genome phylogenies indicated that these isolates were related but not identical clones derived from a single recent contamination event, this pattern is more consistent with repeated selection of pre-adapted lineages than with direct observation of active evolution. In addition, because the present study was observational in design, the temporal patterns reported here should be interpreted as enrichment and persistence of pre-adapted lineages rather than direct evidence of *de novo* evolution. Longitudinal whole-genome sequencing of same-individual isolates collected across time points would be required to evaluate active evolutionary change more rigorously. Importantly, the discrepancy between the high metagenomic abundance of *C. acnes* and its failure to be recovered in culture highlights a key limitation of the present study. Without viability-directed assays such as PMA-qPCR or live/dead staining, the metagenomic detection of *Cutibacterium* should not be interpreted as evidence of an active resistome or ongoing physiological activity. Therefore, the metagenomic signal assigned to *C. acnes* is more conservatively interpreted here as DNA-based detection requiring further validation, rather than evidence of viable or metabolically active bacteria. A further limitation of the present culture-dependent workflow is that it was not specifically optimized for lipid-dependent and fastidious anaerobes such as *Cutibacterium*. More specialized isolation strategies, including lipid-supplemented media and reinforced anaerobic media, may improve recovery of genetically detected but uncultured taxa in future studies. Accordingly, the failure to isolate *Cutibacterium* in the present study should not be interpreted as definitive evidence of absence, but rather as a result that may reflect both biological and methodological constraints.

To maintain these costly genes, colonizing bacteria evolved an exquisitely efficient “four-pillar” functional physical linkage (BMRG-ARG-VF-MGE). Our contig-level matrix reveals that co-integrating heavy metal efflux, biocide tolerance, and clinical antibiotic resistance genes (ARGs) into massive contiguous cassettes is the most economical strategy for ensuring dual environmental and host survival. Strikingly, we observed similar plasmid backbones and large metal-resistance-associated modules across independent individuals. These recurrent genomic configurations are consistent with the possibility that shared anthropogenic pressures may favor the persistence or enrichment of related resistance-associated architectures across hosts. Because the metagenomic cohort was limited to eight samples, the inferred co-occurrence relationships should be interpreted cautiously. These associations are better viewed as exploratory patterns that may inform future validation, rather than definitive evidence of stable ecological or functional linkage.

This pattern of co-selection may pose a potential challenge to skin microbiome homeostasis. Notably, the “ESBL-biocide co-selection module” suggests that grooming biocides may contribute to the persistence of *Klebsiella* lineages carrying extended-spectrum beta-lactamase genes such as *bla*_SHV_. Alarmingly, our metagenomic profiling further detected reads assigned to *mcr-* and *tet*(X)-like resistance gene variants within the broader community, and targeted PCR confirmed the presence of these sequences in the corresponding samples. However, because no viable carrier isolates were recovered and no phenotypic susceptibility testing was performed, these findings should be regarded as evidence for the presence of these resistance determinants in the shaver-associated microbiome rather than as confirmation of clinically relevant resistance in specific viable strains. Nevertheless, the confirmed presence of these genes suggests that the shaver-associated microbial community may serve as a potential reservoir of transferable resistance determinants, which could still pose a risk through horizontal gene transfer to other bacteria. Because routine shaving inevitably strips corneocytes and inflicts epidermal micro-abrasions, it bypasses the primary immune barrier. If viable pathogens—armed with multidrug efflux pumps, adherence fimbriae, necrotizing toxins, and potentially these last-resort ARGs—breach the skin, they could disrupt local immune homeostasis and trigger intractable skin and soft tissue infections (SSTIs), or precipitate severe healthcare-associated outbreaks akin to previously reported *Serratia* cases (Kim et al., 2020).

While microbial contamination on personal items is already recognized, our study applies an integrated metagenomic and culture-dependent WGS pipeline to move beyond static compositional description and to assess the potential presence of viable opportunistic pathogens in this setting. Although our epidemiological inferences require validation through larger longitudinal cohorts, this mechanism-driven study issues a clear warning. Finally, several limitations of this exploratory cohort should be noted. Factors such as shaver brand, specific blade alloy composition, and individual user hygiene practices (e.g., rinsing frequency and storage humidity) were not strictly controlled. While this introduces baseline heterogeneity, it also accurately reflects the *in situ* complexity of real-world usage. Future larger-scale, stratified studies are warranted to disentangle the precise contributions of these specific physicochemical covariates to the shaver pathobiome. Amid the escalating global AMR crisis, we must advance our defensive lines from traditional clinical settings to high-contact daily anthropogenic niches. Personal care fomites are critical evolutionary relay stations facilitating the high-frequency spillover of environmental resistance genes into human communities. Public health authorities must urgently re-evaluate the long-term risks of biocide-containing products and formulate scientifically targeted disinfection guidelines to intercept the community-acquired transmission of superbugs.

## 5 Conclusion

In conclusion, our integrated metagenomic and culture-dependent investigation suggests that electric shavers may represent a selective microenvironment in which skin-associated bacteria, including opportunistic pathogens, can persist together with resistance- and virulence-associated genetic features. The relentless dual physicochemical pressures of alloy heavy metals and grooming biocides impose a strict ecological bottleneck. This anthropogenic stress drives the independent convergent evolution of opportunistic pathogens, forcing them to assemble standardized “four-pillar” mega-islands that inextricably link environmental tolerance (BMRGs) with clinical ARGs, VFs, and MGEs. The covert establishment of these viable superbugs—coupled with the detection of “last-resort” resistance determinants—poses a hidden but imminent threat to human skin homeostasis, providing a direct conduit for community-acquired infections via routine micro-abrasions. Ultimately, this study underscores the urgent necessity to expand proactive AMR surveillance into routine anthropogenic micro-niches. Embracing a comprehensive One Health approach is critical to re-evaluating the unregulated use of household biocides and mitigating the silent spillover of superbugs from daily personal care devices into the broader human population.

## Data Availability

The sequence data presented in the study are publicly available. These data can be found at: https://www.ncbi.nlm.nih.gov/, accession number PRJNA1428499.
