# Supplementary material for: Integrated metagenomic and culture-dependent profiling reveals electric shavers as selective reservoirs for multidrug-resistant opportunistic pathogens
Source: Front Microbiol. 2026 May 7;17:1839764. doi: 10.3389/fmicb.2026.1839764 (PMC13190610; doi:10.3389/fmicb.2026.1839764)
Supplement: Supplementary file 2 [file Table_2.DOCX]

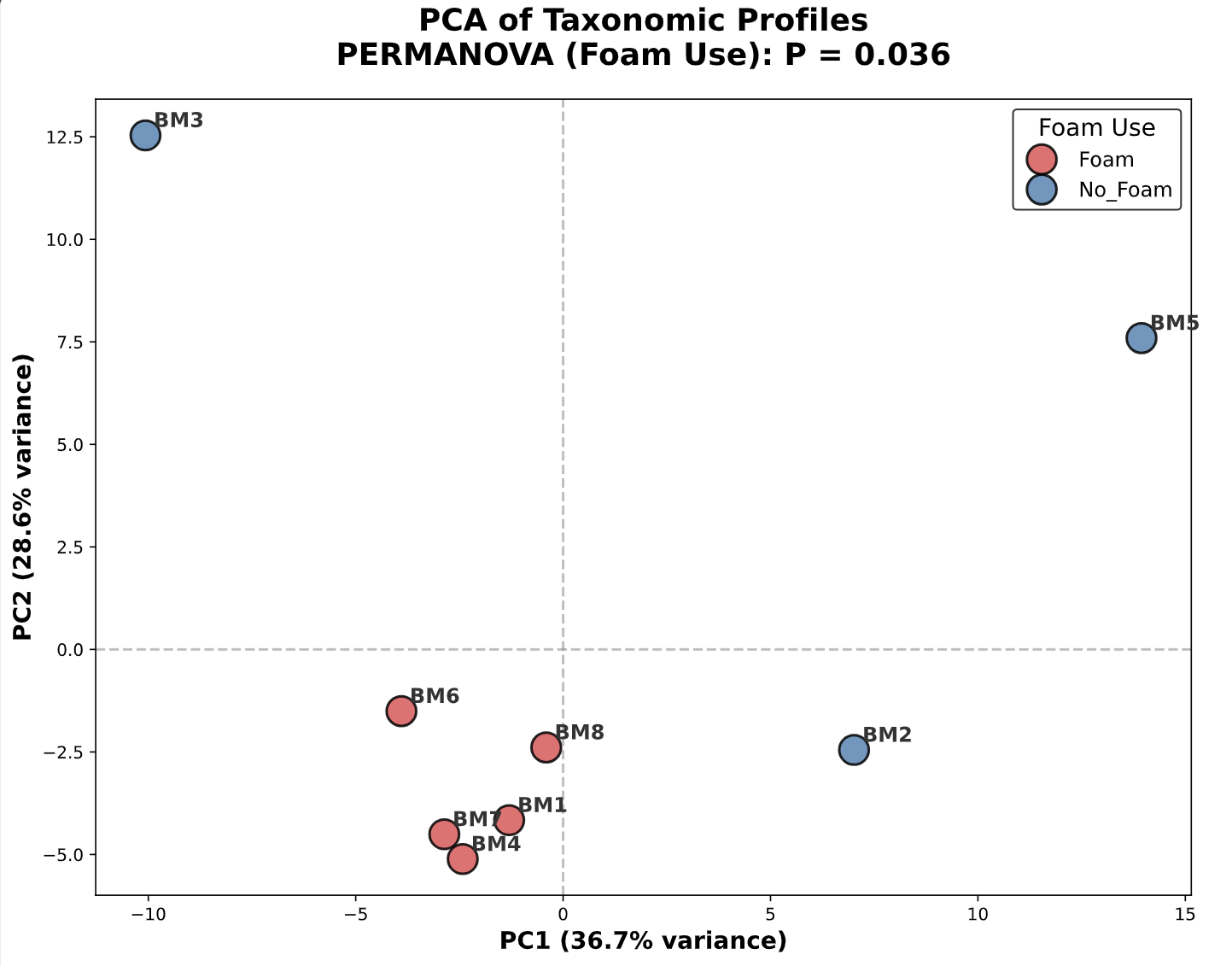


**Fig. S1.** Beta diversity and structural divergence of the mature shaver microbiomes driven by grooming habits. Principal Coordinate Analysis (PCoA) plot based on Bray-Curtis dissimilarity matrices, illustrating the compositional clustering of the Day 21 microbial communities (BM1–BM8). The spatial distribution reveals high inter-individual heterogeneity tightly linked to specific physicochemical constraints.


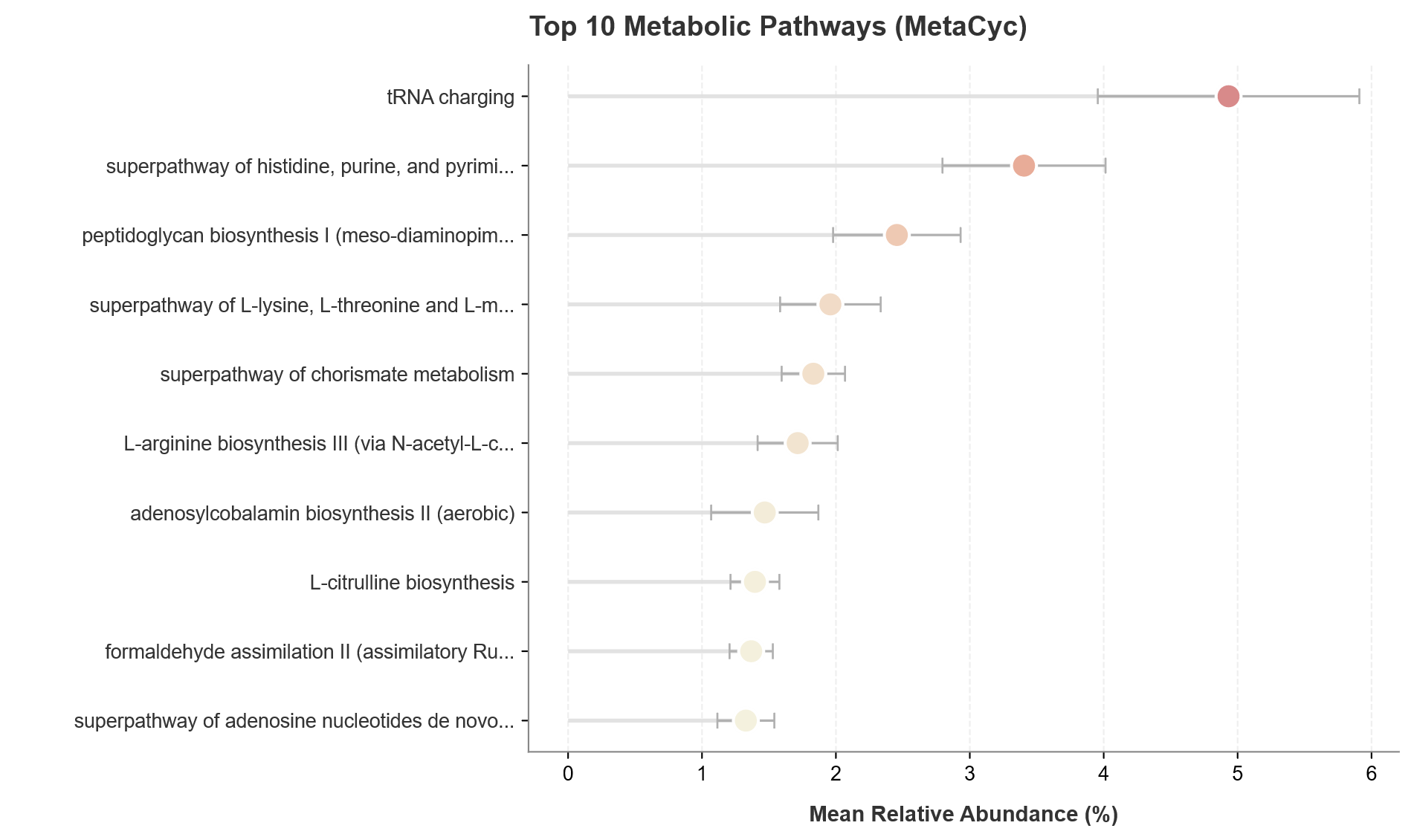


**Fig. S2.** Dominant metabolic framework of the mature shaver microbiome. Bar chart illustrating the top 10 most abundant MetaCyc metabolic pathways, ranked by overall sequence counts across the Day 21 metagenomes. The functional landscape reflects a community under severe physical and nutritional duress.


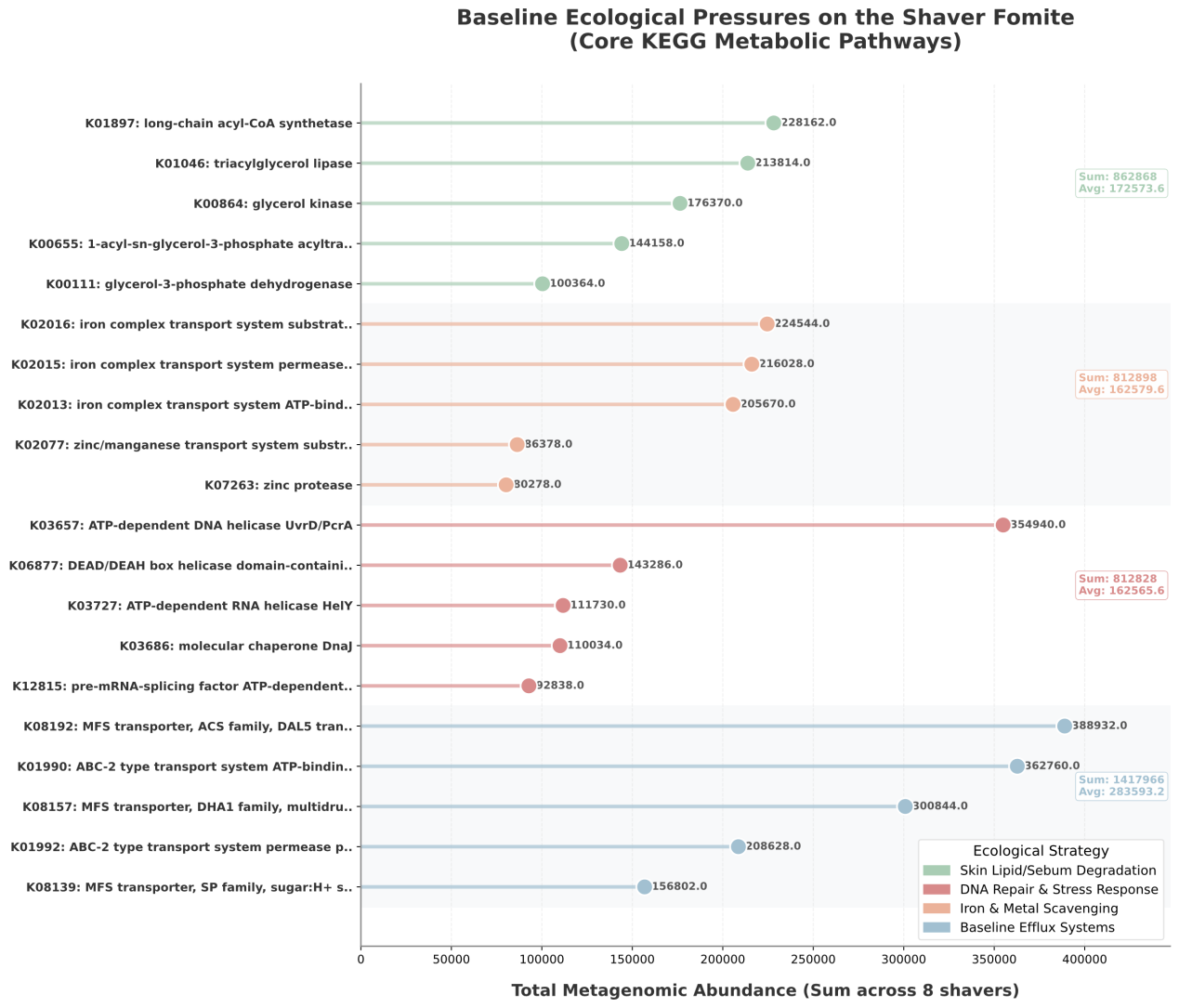
**Fig. S3.** KEGG ortholog enrichment highlights a transporter-heavy and stress-responsive physiological baseline. Bar chart detailing the most highly abundant KEGG Orthologs (KOs) across the mature (Day 21) shaver microbiomes. The functional profile is overwhelmingly dominated by membrane transport and stress-response machinery, reflecting the severe physicochemical selective pressures of the niche. The profound enrichment of MFS and ABC transporters underscores a community-wide reliance on universal efflux systems to continuously expel accumulated toxins, including alloy-derived heavy metals and residual biocides. Furthermore, the high abundance of specific iron-complex transporters (e.g., K02016) indicates intense resource scavenging and competition on the nutrient-depleted metallic surface. The concurrent prevalence of DNA repair helicases (e.g., K03657) highlights the ongoing mitigation of oxidative and chemical DNA damage induced by the extreme micro-environment. Together, these highly conserved functional orthologs establish the essential physiological foundation required for robust colonization.


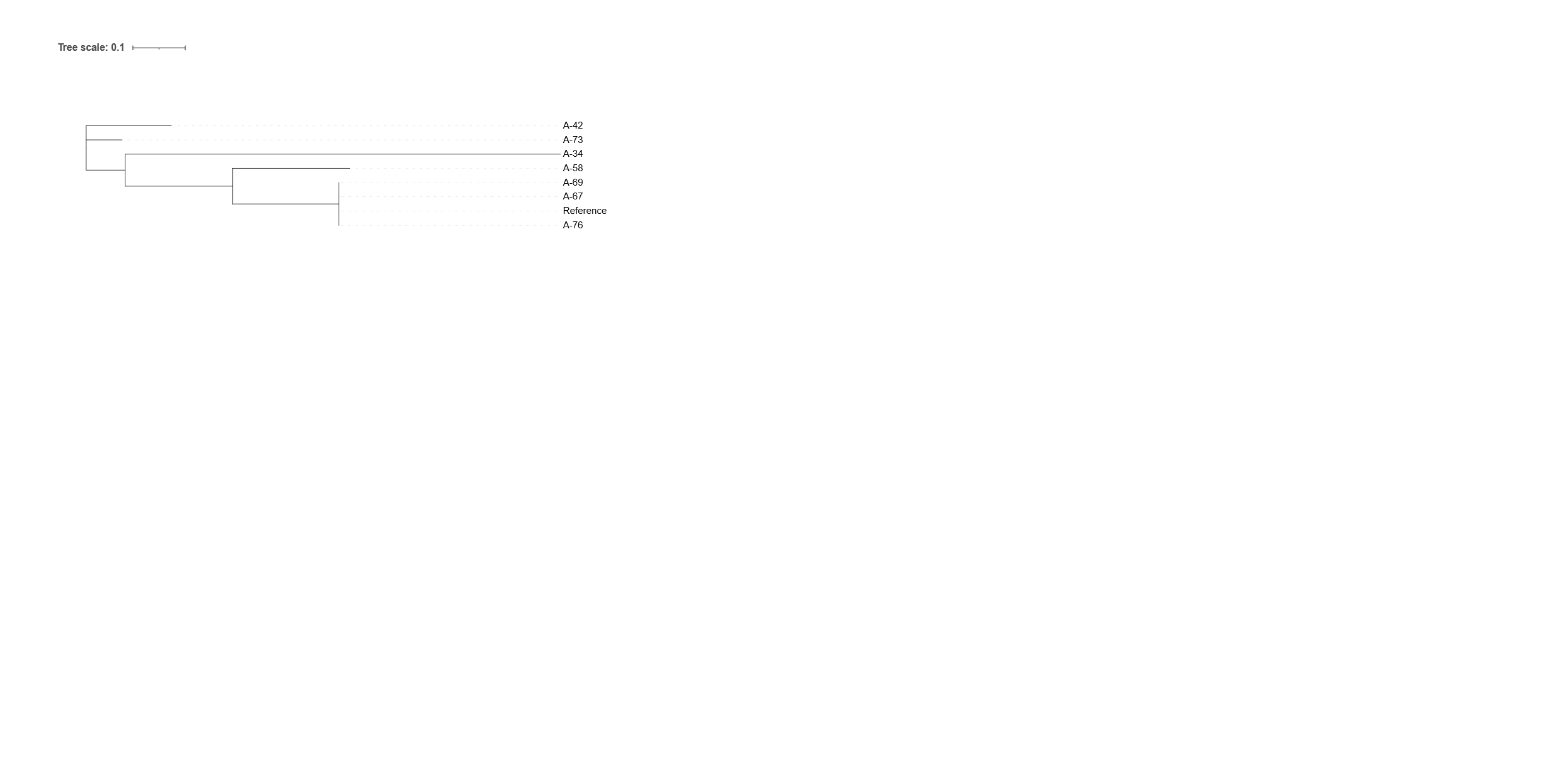


**Fig. S4.** High-resolution core-genome phylogenetic tree of *K. pneumoniae* isolates. The tree was constructed based on a core-genome single nucleotide polymorphism (SNP) alignment. The horizontal axis represents genetic distance, denoted as substitutions per polymorphic site (rather than whole-genome substitution rates), due to the exclusion of invariant regions during tree construction. The branch lengths and the underlying SNP divergence (up to 306 core SNPs) visually corroborate the genetic distinctness of these isolates. This confirms they do not stem from a single, recent cross-contamination event, despite sharing strictly conserved multidrug-resistance mega-islands.
